# Acute Necrotizing Pancreatitis following Long-Term Antipsychotic Use

**DOI:** 10.1155/2021/7891017

**Published:** 2021-08-30

**Authors:** David T. Liebers, Adaora Ofomata, Ryan Badolato, Emily Mills, Pantea Farahmand

**Affiliations:** ^1^NYU Langone Department of Psychiatry, One, 8, Park Ave, New York, NY 10016, USA; ^2^NYU Grossman School of Medicine, 550 1st Avenue, New York, NY 10016, USA; ^3^Hassenfeld Children's Hospital at NYU Langone, 462 1st Avenue 21st floorFloor, New York, NY 10016, USA; ^4^Bellevue Hospital Center, 462 1st Avenue 21st floorFloor, New York, NY 10016, USA

## Abstract

*Introduction*. Psychiatrists commonly use antipsychotic medications in the treatment of psychotic and mood disorders. A rare but known side effect of atypical antipsychotics is acute pancreatitis. Most cases of antipsychotic-induced pancreatitis occur within six months of initiation. The mechanism believed to cause this reaction is hypertriglyceridemia. Here, we present a unique case of antipsychotic-induced pancreatitis that deviates from previous cases in the time to onset of the pancreatitis and the mechanism of presentation. *Case Presentation*. We present a case of a patient with treatment-resistant schizophrenia managed for over a decade on olanzapine and haloperidol. Twelve years after stabilization on this medication regimen, the patient developed acute pancreatitis, which after extensive medical workup was attributed to his psychotropic medications. We review his medical and psychiatric history, his medical course and workup during the episode of pancreatitis, and review recommendations for patients at risk for antipsychotic-induced pancreatitis based on this case and the current literature. *Discussion*. This case illustrates that acute pancreatitis can occur long after the initiation of antipsychotic medications and may be mediated by mechanisms other than hypertriglyceridemia. While there are reports of antipsychotic-induced psychosis occurring within months, and in a limited set of cases, years, after medication initiation, the twelve-year time interval in the present case is by far the longest duration of an antipsychotic precipitating this adverse event recorded in the literature. This case highlights that although exceedingly rare, prescribers should be aware of the risk for drug-induced pancreatitis in patients stable on antipsychotic medications.

## 1. Introduction

Psychiatrists commonly use antipsychotic medications in the treatment of psychotic and mood disorders. A rare but known side effect of atypical antipsychotics is acute pancreatitis [1]. Drug-induced pancreatitis (DIP) is responsible for an estimated 0.1%-2% of all acute pancreatitis cases [2]. Acute pancreatitis ranges in severity from mild inflammation to multiple organ failure and sepsis. A potential consequence of recurrent attacks of pancreatitis is that the tissue within the pancreas may necrose and later become infected leading to acute necrotizing pancreatitis, a condition associated with significant mortality and morbidity [3].

Clozapine, olanzapine, and quetiapine have been implicated in the majority of antipsychotic-related pancreatitis cases [1]. An analysis of the Food and Drug Administration's MedWatch surveillance system revealed that 63% of reported cases of pancreatitis attributed to olanzapine had 6 months or less of drug exposure before the development of pancreatitis [4]. To our knowledge, this was the first report describing a case of acute pancreatitis following long-term olanzapine therapy.

Olanzapine is an atypical antipsychotic that primarily functions as a serotonin-dopamine receptor antagonist. It was modeled similarly to clozapine and approved for psychiatric use in 1996 [5]. A number of case reports have since been released, showing associations between acute pancreatitis and olanzapine therapy. In this article, we describe a patient with schizophrenia who developed acute necrotizing pancreatitis following long-term use of olanzapine in conjunction with haloperidol.

## 2. Case Presentation

Mr. C, a 42-year-old African American man with a past psychiatric history of schizophrenia and past medical history of childhood asthma, hypothyroidism, and hyperlipidemia, presented to a large, academically-affiliated medical center with acute, severe epigastric pain accompanied by several episodes of bilious emesis. On arrival to the emergency room, the patient was afebrile and hemodynamically stable. Computed tomography of the abdomen demonstrated extensive acute pancreatitis, complicated by extensive necrosis, and a multiloculated fluid collection replacing most of the body and proximal tail of the pancreas, measuring 20 cm by 3.2 cm. Initial laboratory testing was notable for lactic acidosis. During the hospitalization, the patient was found to have *P. anaerobius* bacteremia, developed acute hypoxic respiratory failure, and was transferred to the intensive care unit (ICU). The patient subsequently required multiple hospitalizations for endoscopic drainage of the fluid collection and pancreatic stent placement.

Prior to the development of acute necrotizing pancreatitis, the patient had longstanding and complicated history of psychopharmacology for the treatment of schizophrenia. His psychotic illness was refractory to the treatment with multiple typical and atypical antipsychotics, including a trial of clozapine that was ceased due to the development of neutropenia. After a series of failed antipsychotic trials, the patient was eventually stabilized for twelve years on a regimen of haloperidol 10 mg daily, haloperidol decanoate 100 mg every four weeks, and olanzapine 20 mg daily. The patient developed hypertriglyceridemia eight years prior to the development of pancreatitis and was treated with 500 mg docosahexaenoic acid (DHA) and 500 mg eicosapentaenoic acid (EPA) with subsequent improvement in his triglyceride levels. Of note, eight months prior to the development of pancreatitis, the patient's triglyceride level was 162 mg/dL (greater than 200 mg/dL is considered high). On repeat evaluation, one month prior to admission, the patient's triglyceride level was 185 mg/dL. The patient had persistently elevated hemoglobin A1c levels (HbA1c) in the prediabetic range, between 5.7% and 6.6% for the past ten years (4%-5.6% is considered normal). On initial evaluation, during the above hospitalization, his HbA1c remained in that range at 6.2%, reflective of continued pancreatic dysfunction potentially due to atypical antipsychotic treatment.

Common etiologies of acute pancreatitis were considered, though the patient did not have overt triglyceride dysfunction, acute change in HbA1c, gallstones, or recent alcohol or other substance use. An infectious etiology was ruled out as the patient neither showed signs of infection nor met the Systemic Inflammatory Response Syndrome (SIRS) criteria [6]. The lactic acidosis on admission was believed to be secondary to pancreatic damage and has been shown to be a predictor of severity of disease [7]. While the patient had *Peptostreptococcus* bacteremia, this was later in the hospital course and thus determined to be a consequence, rather than a cause, of pancreatitis. The patient also did not have any recent abdominal instrumentation or reported family history of pancreatitis. Given the absence of other significant risks factors for pancreatitis, iatrogenic causes were considered.

Of the patient's medications, olanzapine was identified as the most likely causative agent for the development of pancreatitis. Thus, olanzapine was discontinued and his dose of haloperidol decanoate was increased to 200 mg every four weeks. On follow-up, the patient had developed insulin-dependent diabetes mellitus, though the pancreatitis itself had not progressed further. Ten months after the initial event, the patient's HbA1c was 12.5% and he was hospitalized several times for complications of hyperglycemia. Repeat abdominal imaging demonstrated extensive loss of pancreatic tissue, replaced by fluid collections and chronic inflammation.

## 3. Discussion

The case presented here illustrates a rare but life-threatening reaction to antipsychotic medications. Previously reported cases of olanzapine-related DIP occurred soon after the initiation of treatment and in the setting of significantly elevated triglycerides [8–11]. This case illustrates that acute pancreatitis can occur long after the initiation of olanzapine and may be mediated by mechanisms other than hypertriglyceridemia. While there are reports of DIP attributed to olanzapine occurring months, and in a limited set of cases, years, after medication initiation, the twelve-year time interval in the present case is by far the longest duration of olanzapine therapy precipitating this adverse event recorded in the literature [12, 13]. In cases where hypertriglyceridemia was posited as mediating DIP, triglyceride levels increased into the thousands of mg/dL, whereas in this case, triglycerides remained only slightly above normal range [9, 14].

One of the chief problems in assessing adverse events is establishing a causal relationship between the drug and clinical outcome. Naranjo et al. [15] addressed this with an adverse drug reaction probability questionnaire to help standardize research. Based on this widely used scale, in this case, the likelihood of olanzapine as causal agent is ‘probable' and of greater probability than the alternative hypothesis of haloperidol as a causal agent, since progression of disease stabilized with removal of olanzapine even with an increased dose of haloperidol.

Mr. C's particularly severe and unusual case of DIP serves as a reminder to clinicians prescribing antipsychotics to discuss early warning signs of pancreatitis with patients at potentially elevated risk for this outcome (due to history of alcohol use disorder, hyperlipidemia, or treatment with other medications associated with pancreatitis) and to revisit this discussion throughout the course of treatment. Clinicians could also consider adding amylase and lipase to the annual blood work of patients on prolonged courses of antipsychotics.

## Data Availability

Data is available for access and can be requested by contacting Pantea Farahmand for deidentified information.

## Abbreviations

DIP: Drug-induced pancreatitis
ICU: Intensive care unit
DHA: Docosahexaenoic acid
EPA: Eicosapentaenoic acid
HbA1c: Hemoglobin A1c levels.
